# Assembly properties of bacterial actin MreB involved in *Spiroplasma* swimming motility

**DOI:** 10.1016/j.jbc.2023.104793

**Published:** 2023-05-05

**Authors:** Daichi Takahashi, Makoto Miyata, Ikuko Fujiwara

**Affiliations:** 1 Graduate School of Science, Osaka Metropolitan University, Osaka, Japan; 2 The OMU Advanced Research Center for Natural Science and Technology, Osaka Metropolitan University, Osaka, Japan; 3 Department of Materials Science and Bioengineering, Nagaoka University of Technology, Nagaoka, Niigata, Japan

**Keywords:** actin, bacteria, cytoskeleton, ATPase, electron microscopy, cell motility, polymerization dynamics, bundle formation, Mollicutes, Mycoplasma

## Abstract

Bacterial actin MreB forms filaments composed of antiparallel double-stranded units. The wall-less helical bacterium *Spiroplasma* has five MreB homologs (MreB1–5), some of which are involved in an intracellular ribbon for driving the bacterium’s swimming motility. Although the interaction between MreB units is important for understanding *Spiroplasma* swimming, the interaction modes of each ribbon component are unclear. Here, we examined the assembly properties of *Spiroplasma eriocheiris* MreB5 (SpeMreB5), one of the ribbon component proteins that forms sheets. Electron microscopy revealed that sheet formation was inhibited under acidic conditions and bundle structures were formed under acidic and neutral conditions with low ionic strength. We also used solution assays and identified four properties of SpeMreB5 bundles as follows: (I) bundle formation followed sheet formation; (II) electrostatic interactions were required for bundle formation; (III) the positively charged and unstructured C-terminal region contributed to promoting lateral interactions for bundle formation; and (IV) bundle formation required Mg^2+^ at neutral pH but was inhibited by divalent cations under acidic pH conditions. During these studies, we also characterized two aggregation modes of SpeMreB5 with distinct responses to ATP. These properties will shed light on SpeMreB5 assembly dynamics at the molecular level.

MreB belongs to the actin superfamily and is conserved in the bacterial kingdom (1). It possesses a canonical actin fold, which is composed of four subdomains (IA, IB, IIA, and IIB) (2, 3, 4, 5, 6). MreB molecules polymerize into antiparallel double-stranded filaments and undergo repeat polymerization and depolymerization, depending on ATP (2). The MreB filaments bind to the cell membrane *via* their membrane-binding sites in subdomain IA (3, 7) and form an elongasome complex, which is a bacterial cell wall (peptidoglycan) synthesis complex during the growth phase (8).

Although one of the most recognized roles of MreB is elongasome formation, several bacteria use MreBs for other cellular activities (4, 9, 10, 11, 12, 13). *Spiroplasma* belongs to the class Mollicutes and is characterized as a wall-less helical cell (14, 15). Each *Spiroplasma* species possesses five classes of MreBs (MreB1–5) (16, 17), and at least three of them form an intracellular helical ribbon structure with a *Spiroplasma*-specific cytoskeletal protein fibril (18, 19, 20, 21). The ribbon helicity is switched possibly by dynamics related to the polymerization and depolymerization of MreB2 and/or MreB5, and helicity switching is transmitted along the ribbon structure (22). These helicity-switching dynamics generate a propulsive force for a cell to swim in a liquid (23, 24, 25). This swimming is completely different from the conventional types of bacterial motility, such as flagellar and pili motility (26).

While the unit of the MreB filament is a double strand, the majority of the reported MreBs also have higher-ordered structures. Previous studies have shown that the MreBs of various species form sheet structures (2, 6, 27, 28, 29, 30). In the ribbon structure of *Spiroplasma* cells, MreB(s) form sheets and are located between two bundles of fibril filaments and/or between a fibril bundle and the cell membrane (20, 21). *Spiroplasma eriocheiris* MreB5 (SpeMreB5), an essential MreB for *Spiroplasma* swimming (4, 10, 11), forms sheet structures *in vitro*, which include an antiparallel double-stranded filament at one edge of the sheet. The other protofilaments in the sheet are aligned parallel to adjacent protofilaments (2). In contrast, many proteins in the actin superfamily form bundles under several *in vitro* conditions (31). Actin forms bundles in the presence of 10 to 50 mM divalent cations. These bundle formations depend on the negatively charged nature of the actin filament surfaces (31, 32, 33, 34). Several walled-bacterial MreBs have been reported to form bundles, the formation efficiencies of which depend on pH, ionic strength, and divalent cations (27, 28, 29, 30, 35). Analyses of the sheet and bundle formation processes of cytoskeletal proteins are important for understanding their behaviors at the molecular level. However, the limited experimental conditions for MreB hamper the full understanding of its sheet- and bundle-formation processes. Moreover, bundle formation of *Spiroplasma* MreBs are poorly characterized.

In this study, we analyzed the assembly property of SpeMreB5. Electron microscopy (EM) under various conditions revealed bundle formation of SpeMreB5. Light scattering and sedimentation assays revealed the molecular properties of bundles. These findings provide clues to understand the properties of SpeMreB5. During these studies, we found two aggregation modes of SpeMreB5 that showed distinct responses to ATP.

## Results

### SpeMreB5 forms double-stranded filaments and bundles other than sheets

**Figure 1:**
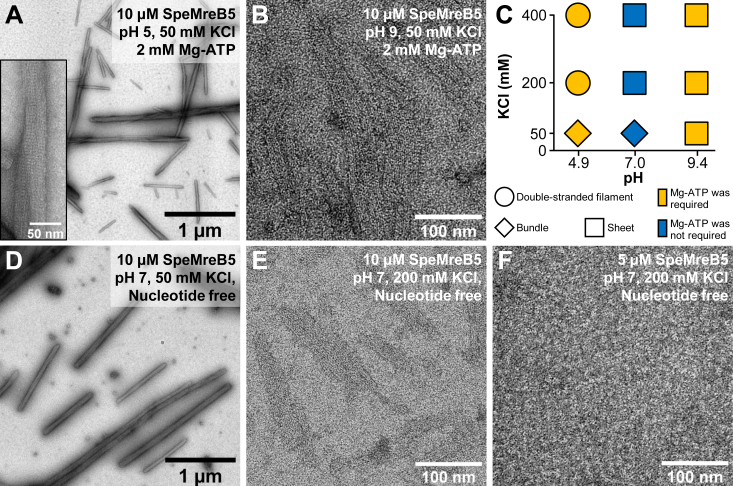
**pH and ionic strength dependence of assembled SpeMreB5 structures.***A* and *B*, negative-staining EM images of 10 μM SpeMreB5 polymerized in the presence of 2 mM Mg-ATP with 50 mM KCl at pH (*A*) 5 and (*B*) 9. A magnified image of the bundle is shown in the inset of *A*. *C*, phase diagram of SpeMreB5 filament structures at 10 μM protein concentration over a range of pH and KCl concentrations. Filament structures in each condition are indicated by symbols as follows: double-stranded filament (*circle*), sheet (*square*), and bundle (*diamond*). The color of the symbol indicates whether Mg-ATP is required (*orange*) or not (*blue*). *D*–*F* negative-staining EM images of (*D*–*E*) 10 and (*F*) 5 μM SpeMreB5 polymerized in the absence of nucleotides at pH 7 with (*D*) 50 and (*E* and *F*) 200 mM KCl. Scale bars are indicated in each panel.

We expressed and purified monomeric SpeMreB5 as the fusion with a 6 × His-tag, as described (2). We first polymerized 10 μM SpeMreB5 by adding 2 mM Mg-ATP under three different pH conditions (50 mM CH_3_COOH-KOH pH 4.9, HEPES-KOH pH 7.0, and CHES-KOH pH 9.4 as representatives of acidic, neutral, and basic pH conditions, respectively) over a range of KCl concentrations (50, 200, and 400 mM) and observed using negative-staining EM (Figs. 1, *A* and *B*, S1, *A*–*G*). At pH 7 and 200 mM KCl, SpeMreB5 formed sheet structures, as observed in our previous study (Fig. S1*D*) (2). Sheets were also formed at pH 7 with 400 mM KCl and at pH 9 (Figs. 1*B*, S1, *E*–*G*). Among the nine tested conditions, sheet formation was dominant for SpeMreB5 (Fig. 1, *B* and *C*). Interestingly, we found filamentous structures other than sheets under several conditions (Fig. 1*C*). At pH 5 and 7 with 50 mM KCl, SpeMreB5 formed bundle structures in which protofilaments were packed (Figs. 1*A* and S1*C*). The bundle length reached several micrometers. These structures have also been reported in *Thermotoga maritima* MreB (TmMreB) and *Escherichia coli* MreB (EcMreB) (29, 30). In contrast, at pH 5 with 200 and 400 mM KCl, SpeMreB5 formed double-stranded filaments (Fig. S1, *A* and *B*), which has also been reported in *S. eriocheiris* MreB3 (SpeMreB3) and *Caulobacter crescentus* MreB (2, 5). We also performed the same experiments on SpeMreB3, which was purified as a monomer in our previous study (2). At pH 7, SpeMreB3 formed double-stranded filaments regardless of the KCl concentration, as in our previous study (Fig. S1, *H*–*J*) (2). However, filamentous structures were not observed under the other pH conditions (Fig. S1, *K*–*L*), which did not allow us to validate the sheet and bundle formation of SpeMreB3.

We also performed negative-staining EM in the absence of Mg-ATP. SpeMreB5 at pH 5 and 9 and SpeMreB3 with the concentration of 10 μM did not form filamentous structures, as confirmed in our previous study (Fig. S1, *M*–*Q*) (2). Interestingly, 10 μM SpeMreB5 (at pH 7) polymerized even in the absence of Mg-ATP (Fig. 1, *D* and *E*). The experimental conditions of this study differ from those of our previous study in protein concentrations (5 μM in the previous study and 10 μM in this study) and buffers (20 mM Tris-HCl pH 7.5 in a previous study and 50 mM HEPES-KOH pH 7.0 in this study) (2). In the HEPES buffer, 5 μM SpeMreB5 formed filamentous structures in the presence of Mg-ATP (Fig. S1*R*) but not in the absence of Mg-ATP (Fig. 1*F*). These results indicate that ATP promotes SpeMreB5 polymerization. In contrast, 10 μM SpeMreB5 polymerized in the absence of Mg-ATP in the Tris buffer used in the previous study (Fig. S1*S*) (2). These results indicate that SpeMreB5 with a concentration of approximately 10 μM or more polymerizes even without nucleotides at neutral pH.

### Surface potential maps of SciMreB5 over the range of pH

**Figure 2:**
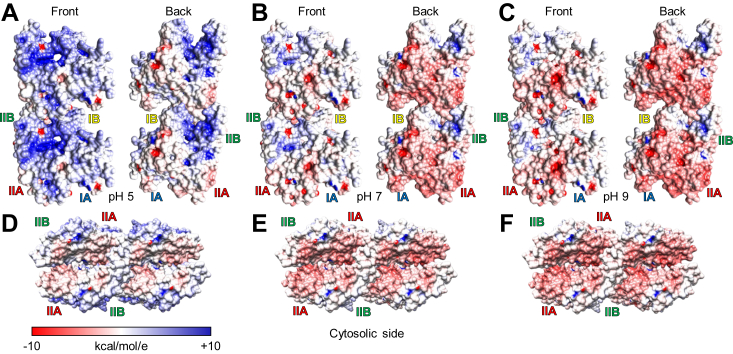
**Surface potential maps of SciMreB5.** The interprotofilament interaction surface for the antiparallel filament formation is defined as “back,” and the opposite side is defined as “front.” The coulombic electrostatic potential is indicated by a color gradient from blue (10 kcal/mol/e) to red (−10 kcal/mol/e); namely, blue, white, and red regions indicate positively charged, uncharged, and negatively charged regions, respectively. *A*–*C*, potential maps of the protofilaments with two subunits of SciMreB5 AMPPNP (PDB: 7BVY) at pH (*A*) 5, (*B*) 7, and (*C*) 9. The four subdomains are labeled for the lower subunits in the protofilament. *D*–*F*, potential maps on the cytosolic side of the double-stranded filament model of SciMreB5 AMPPNP (PDB: 7BVY) at pH (*D*) 5, (*E*) 7, and (*F*) 9. The structural model was created by fitting four SciMreB5 AMPPNP molecules to each subunit of a double-stranded filament structure of *Caulobacter crescentus* MreB (PDB: 4CZJ). The positions of facing subdomains (IIA and IIB) are labeled for the left-side subunits.

To discuss the atomic basis of sheet and bundle formation, we calculated the surface potential maps of a crystal structure of the MreB5 protofilament reported previously (*Spiroplasma citri* MreB5 [SciMreB5]) (PDB:7BVY) (4), which is 87.5% identical to SpeMreB5 and has a net charge +1 higher than that of SpeMreB5 (Fig. S2*A*) (2)), at pH 5, 7, and 9 (Fig. 2, *A*–*C*). In this study, we call the interprotofilament interaction surface for the antiparallel filament formation “back” and the opposite side “front.” We also fitted these structures to the antiparallel double-stranded filament of *Caulobacter crescentus* MreB (PDB:4CZJ), in which the crystal structure has been reported (5), to validate potential maps of filament sides (Figs. 2, *D*–*F* and S2, *B*–*D*). We call the side for subdomains IA and IB “membrane side” and the opposite side “cytosolic side” as MreBs bind to the membrane *via* two consecutive hydrophobic residues, an N-terminal amphipathic helix, and/or a positively charged C-terminal tail all at subdomain IA (3, 7). For comparison, we calculated the surface potential maps of SpeMreB3 (PDB:7E1G), TmMreB (PDB:1JCG), and EcMreB (modeled by AlphaFold2 (36)) at pH 7 (Fig. S2, *E*–*G*), where EM studies have been conducted (2, 29, 30, 35). Of note, we excluded the membrane sides from the following discussion, as the terminal regions of SciMreB5 and SpeMreB3 with around 20 residues that should occupy the membrane sides were not visualized (Fig. S2, *B*–*E*).

The SciMreB5 protofilament at pH 5 was mostly positively charged on the back and front sides, whereas its cytosolic region was surrounded by weakly negatively charged regions (Fig. 2, *A* and *D*). The potential map of the SciMreB5 protofilament at pH 7 differed strikingly from that at pH 5 (Fig. 2, *A*, *B*, *D* and *E*). The back side of the protofilament was mostly surrounded by negatively charged regions. The charge on the front side remained weakly positive, whereas that of the subdomain IIA moiety became weakly negative (Fig. 2*B*). The negative charges on the cytosolic regions at pH 7 were stronger than those at pH 5 (Fig. 2, *D* and *E*). These features are common to the potential maps of TmMreB and EcMreB protofilaments (Fig. S2, *F* and *G*) and different from that of the SpeMreB3 protofilament, in which the front and back sides and the cytosolic region are positively charged (Fig. S2*E*). The overall surface charge distribution of the SciMreB5 protofilament at pH 9 was slightly different from that at pH 7 (Fig. 2, *B*, *C*, *E* and *F*). A difference in the distributions is found at the subdomain IIA moiety of the front side, in which the negatively charged region becomes wider at pH 9 than at pH 7. Moreover, the negative charge on the overall structure became slightly stronger. These differences in the surface potential probably caused the various sheet and bundle formation modes of SpeMreB5 under various solution conditions (Fig. 1*C*).

### The bundle formation of SpeMreB5 at neutral pH follows by disaggregation and sheet formation

**Figure 3:**
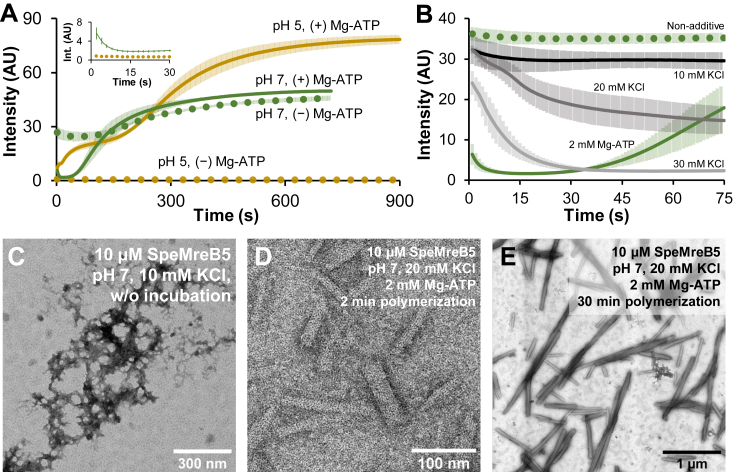
**Dynamics of bundle formation and aggregation disassembly.** For time-course light scattering, data are shown as mean ± SD from three independent measurements. *A*, assembly dynamics of 10 μM SpeMreB5 at pH 5 (ocher) and 7 (green) with 10 mM KCl measured by light scattering. The measurements in the presence of 2 mM Mg-ATP and in the absence of nucleotides are plotted as solid and dotted lines, respectively. The spectra for the first 30 s at pH 7 with Mg-ATP and at pH 5 without Mg-ATP are highlighted in the inset. *B*, initial dynamics of 10 μM SpeMreB5 in the absence of additives (*green dotted line*) and by the addition of 2 mM Mg-ATP (*green solid line*) and 10 (*black*), 20 (*dark gray*), and 30 (*light gray*) mM KCl at pH 7 with the initial KCl concentration of 10 mM measured by light scattering. *C*–*E*, negative-staining EM image of 10 μM SpeMreB5 (*C*) without incubation and (*D*–*E*) polymerized for (*D*) 2 and (*E*) 30 min at pH 7 in the presence of 2 mM Mg-ATP. KCl concentration in *C* was the same as that in *A* and *B* (10 mM), while that in *D* and *E* was 20 mM to obtain the assembly dynamics with a longer lag phase than that with 10 mM KCl (see Fig. S3*G*). Scale bars are indicated in each panel.

To clarify the assembly dynamics of the SpeMreB5 higher-order structures observed under EM (diamonds and squares in Fig. 1*C*), we performed static light scattering assays. The samples were kept on ice at 5 × concentrations prior to the experiments and were measured for the scattering of 650 nm light toward 90° at 25 °C. In time-course measurements, we set the KCl concentration to 10 mM to promote assembly dynamics (Fig. 3*A*). At pH 7, the scattering pattern sequentially transitioned through three states over time after the addition of 2 mM Mg-ATP as follows: (I) the scattering intensity dropped to the background level in the first ∼10 s, (II) the low scattering intensity continued for ∼30 s as the “lag phase,” and (III) the scattering intensity dramatically increased and reached the plateau in approximately 10 min (Fig. 3*A* green solid line). The scattering profile at pH 5 did not have a lag phase and did not show an initial drop in intensity, unlike that at pH 7. Instead, the scattering profile showed two phases, in which the first phase reached a plateau in approximately 2 min and the second phase reached it in approximately 15 min (Fig. 3*A*, ocher solid line). The plateau intensity of the second phase at pH 5 was 1.6 times higher than that at pH 7, probably reflecting the difference in the heterogeneity of higher-order structures including sheets and bundles. The scattering intensity at pH 5 did not increase in the absence of Mg-ATP (Fig. 3*A*, ocher dotted line), indicating that Mg-ATP was a factor that increased the intensity. The initial scattering intensity at pH 7 is high in the absence of Mg-ATP. The intensity at the nucleotide-free condition was unchanged for approximately 3 min and gradually increased to the plateau in approximately 10 min with the intensity similar to that in the presence of Mg-ATP (Fig. 3*A*, green dotted line).

**Figure 4:**
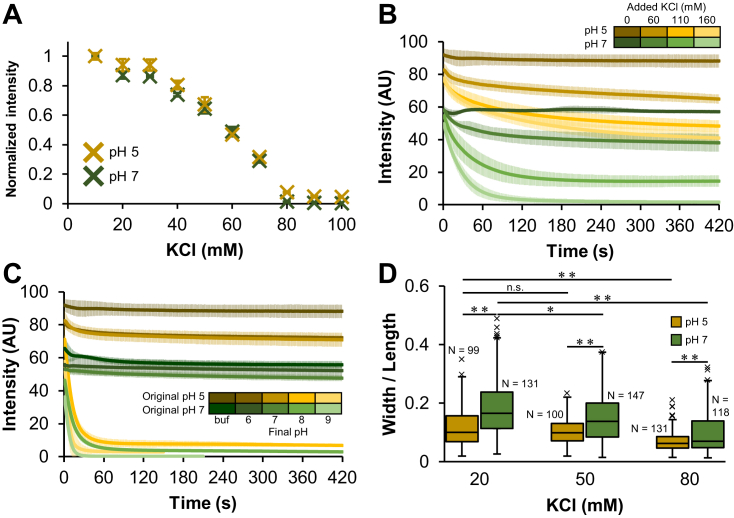
**Electrostatic interaction dependence of SpeMreB5 bundle.** For time-course light scattering, data are shown as mean ± SD from three independent measurements. *A*, normalized steady-state light scattering of 10 μM SpeMreB5 polymerized in the presence of 2 mM Mg-ATP over the range of KCl concentration at pH 5 (ocher) and 7 (*green*). Bars indicate SD from three independent measurements. *B* and *C*, disassembly dynamics of SpeMreB5 bundles measured using light scattering. The bundle solutions were prepared by polymerizing 10 μM SpeMreB5 with 2 mM Mg-ATP in buffers of 10 mM CH_3_COOH-KOH pH 4.9 (ocher scaled colors) and 10 mM HEPES-KOH pH 7.0 (green scaled colors) with 40 mM KCl. The measurements in which the buffer composition was unchanged are indicated with the darkest colored lines. *B*, disassembly was induced by increasing KCl concentration into 100 (second darkest colored line in each color scale), 150 (second lightest colored), and 200 (lightest colored) mM. *C*, disassembly was induced by changing the buffer pH into 6 (second darkest colored line in each color scale), 7 (medium colored), 8 (second lightest colored), and 9 (lightest colored) by adding 50 mM MES-KOH pH 6.0, HEPES-KOH pH 7.0, HEPES-KOH pH 8.1, and CHES-KOH pH 9.4, respectively. *D*, aspect ratio of bundles polymerized by 10 μM SpeMreB5 with 2 mM Mg-ATP at pH 5 (ocher) and 7 (*green*) over a range of KCl concentrations. All detectable bundles were measured for each micrograph and accumulated until approximately 100 data were collected. Sample numbers of each condition are indicated on each box. Values over the upper fence (75th percentile + 1.5 × (75th percentile – 25th percentile)) are defined as outliers and plotted as black crosses. Symbols indicate *p*-value supported by Student’s *t-*test (∗*p* < 0.05, ∗∗*p* < 0.01, and n.s. *p* > 0.05).

**Figure 5:**
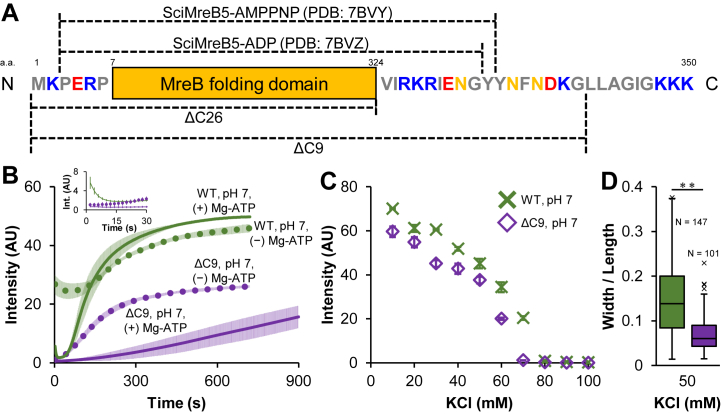
**Assembly dynamics of bundles by the C-terminus-truncated variant of SpeMreB5.***A*, schematics of the SpeMreB5 sequence. The MreB folding domain is defined as the visible region in all the MreB crystal structures reported previously (2, 3, 4, 5, 6) and invisible-flexible loops within them. The residues exterior of the MreB folding domain are shown with *gray*, *orange*, *red*, and *blue* for hydrophobic, nonpolar hydrophilic, acidic, and basic ones, respectively. The regions that are visible in the previously reported crystal structures of SciMreB5 (3, 4) and for SpeMreB5 ΔC9 and ΔC26 variants are indicated above and underneath the schematics, respectively. *B*, assembly dynamics of 10 μM SpeMreB5 WT (*green*, the same traces as those in Fig. 3*A*) and ΔC9 variant (*purple*) at pH 7 with 10 mM KCl measured using light scattering. The measurements in the presence of 2 mM Mg-ATP and in the absence of nucleotides are plotted as solid and dotted lines, respectively. Data are shown as mean ± SD from three independent measurements. The spectra of the first 30 s are highlighted in the inset. *C*, steady-state light scattering of 10 μM SpeMreB5 WT (*green*, the same plots as those in Fig. S3*I*) and ΔC9 (*purple*) polymerized in the presence of 2 mM Mg-ATP over a range of the KCl concentration at pH 7. Bars indicate SD from three independent measurements. *D*, aspect ratio of bundles polymerized by 10 μM SpeMreB5 WT (*green*, the same plot as that in Fig. 4*D*) and ΔC9 (*purple*) in the presence of 2 mM Mg-ATP at pH 7 with 50 mM KCl. All detectable bundles were measured for each micrograph and accumulated until approximately 100 data were collected. Sample numbers of each condition are indicated on each box. Values over the upper fence (75th percentile + 1.5 × (75th percentile – 25th percentile)) are defined as outliers and plotted as black crosses. Symbols indicate *p*-value supported by Student’s *t-*test (∗∗*p* < 0.01).

**Figure 6:**
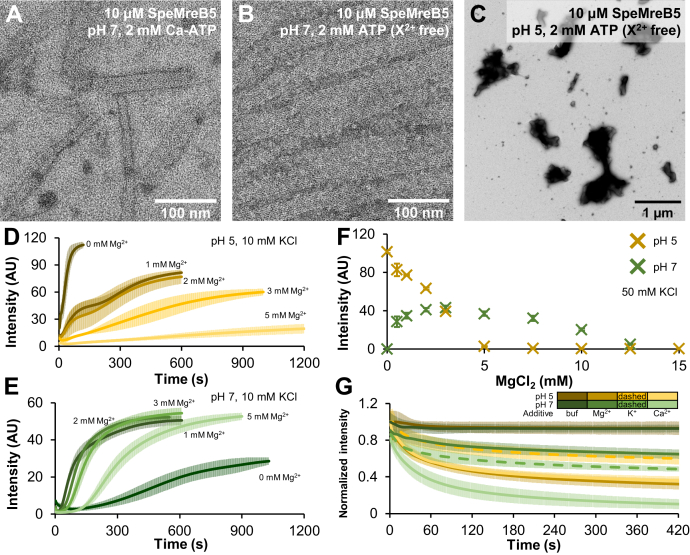
**Divalent cation dependence of SpeMreB5 polymerizationand higher-order structure formation.** For time-course light scattering, data are shown as mean ± SD from three independent measurements. For divalent cation-free conditions, 1 mM EDTA-NaOH pH 8.0 was added to avoid effects from contaminating amounts of multivalent cations. *A*–*C*, negative-staining EM images of 10 μM SpeMreB5 polymerized with varying divalent cation conditions with 50 mM KCl. SpeMreB5 was incubated at pH (*A* and *B*) 7 and (*C*) 5 in the presence of (*A*) 2 mM Ca-ATP and (*B* and *C*) 2 mM ATP (divalent cation-free). Scale bars are indicated in each panel. *D* and *E*, Mg^2+^-dependent assembly dynamics of 10 μM SpeMreB5 at pH (*D*) 5 and (*E*) 7 with 10 mM KCl measured using light scattering. The polymerization was initiated by adding 2 mM ATP with varying concentrations of MgCl_2_ as indicated in the color scales in the panels. *F*, steady-state light scattering of 10 μM SpeMreB5 polymerized with 2 mM ATP at pH 5 (ocher) and 7 (*green*) over a range of MgCl_2_ concentration. KCl concentration was 50 mM constant. Bars indicate SD from three independent measurements. *G*, normalized light scattering traces of disassembly dynamics of SpeMreB5 bundles induced with divalent cations. The bundle solutions were prepared by polymerizing 10 μM SpeMreB5 in the presence of 2 mM Mg-ATP at pH 5 (ocher scaled colors) and 7 (*green* scaled colors) with 50 mM KCl. Disassembly was induced by adding 15 mM MgCl_2_ (second darkest colored solid line), 15 mM CaCl_2_ (lightest colored solid line), and 60 mM KCl (second lightest colored dashed line). Of note, the ionic strengths of these salts are identical assuming the same degree of dissociation (see Experimental procedures). The measurements in which the buffer condition was unchanged are indicated by the darkest colored solid lines.

To clarify the mechanism of the initial intensity drop by adding Mg-ATP at pH 7, we added various salts to SpeMreB5 at pH 7. The initial intensity also decreased to the background level after the addition of 30 mM KCl (Fig. 3*B*), in which the ionic strength is comparable to 2 mM Mg-ATP (approximately 24 mM) assuming the net charge of ATP of −4 (37) and the degree of dissociation of all salts of 1 (see Experimental procedures). This phenomenon was also observed by the addition of various salts with the ionic strength comparable to 2 mM Mg-ATP. However, Mg-ATP caused the most efficient and rapid intensity decrease among the tested reagents (Fig. S3, *A* and *B*). These results indicate that both ionic effect and ATP interaction were related to the initial intensity drop of SpeMreB5 at pH 7. To further characterize the decrease in the initial intensity, we performed the following two experiments. First, we measured the assembly dynamics of SpeMreB5 at pH 7 in the presence of 2 mM Mg-AMPPNP (an unhydrolyzed ATP analogue) or Mg-ADP, instead of Mg-ATP. SpeMreB5 formed bundles at pH 7 in the presence of these ATP analogues (Fig. S3, *C* and *D*). The plateau intensities with Mg-AMPPNP or Mg-ADP were approximately 70% compared with those with Mg-ATP, suggesting that the lower polymerization activity of SpeMreB5 using Mg-AMPPNP and Mg-ADP compared with that using Mg-ATP (2) affected the bundle formation efficiency. SpeMreB5 in the presence of Mg-ADP reached the plateau faster than that in the presence of Mg-ATP and Mg-AMPPNP, probably owing to the lower ionic strength of 2 mM Mg-ADP (approximately 17 mM) than those of 2 mM Mg-ATP and Mg-AMPPNP (approximately 24 mM) assuming the net charge of ADP, ATP, and AMPPNP of −3, −4, and −4, respectively (37), and the degree of dissociation of these salts of 1 (see Experimental procedures). Despite these differences, the scattering profiles composed of three sequential states were common among the three different nucleotide conditions (Fig. S3*E*). Second, we added 2 mM Mg-ATP to the sample that had already reached a plateau in the absence of nucleotides, that is, Mg-ATP addition after SpeMreB5 bundle formation in the absence of nucleotides (Fig. 1*D*). The scattering intensity remained unchanged, even after the addition of 2 mM Mg-ATP (Fig. S3*F*), indicating that bundles were resistant to both the ionic strength increase by the addition of 2 mM Mg-ATP and the ATP interaction. These results indicate that the decrease in the initial intensity of SpeMreB5 at pH 7 was caused by disruption of higher-order structures other than bundles by ionic effects and nucleotide interactions rather than hydrolysis.

To clarify the structural basis of the assembly dynamics, we observed SpeMreB5 in each state of the assembly dynamics. Prior to polymerization at pH 7, aggregating structures of submicrometer sizes were observed instead of filamentous structures (Fig. 3*C*). To visualize the lag phase in the presence of Mg-ATP at pH 7, we increased the KCl concentration to 20 mM, in which the lag phase was approximately 2 min longer than that at 10 mM KCl for ease of handling (Fig. S3*G*). SpeMreB5 in the lag phase formed sheet structures (Fig. 3*D*), together with thin and small bundles of submicrometer lengths (Fig. S3*H*). Bundle structures were formed at the plateau, as was our first EM observation (Figs. 3*E* and S1*C*). These results indicate that SpeMreB5 at neutral pH changes the assembly state in the order of aggregate, sheets, and bundles.

We also observed the first plateau at pH 5 in the presence of Mg-ATP using EM. However, sheet structures were not observed, unlike at pH 7. This result is consistent with our first EM observations, where sheets were not observed at pH 5 (Figs. 1C and S1, *A* and *B*).

### SpeMreB5 bundle formation requires electrostatic interactions

Our light scattering assays were able to measure SpeMreB5 bundle formation (Figs. 3 and S3). Using these assays, we studied the formation mechanism of SpeMreB5 bundles. We first measured the steady-state intensities of SpeMreB5 bundles over a range of ionic strengths (Figs. 4*A* and S3*I*). At both pH 5 and 7, the scattering intensities were mostly constant at 10 to 30 mM KCl, decreased as the KCl concentration increased at 40 to 70 mM KCl, and became less than the detection limit at 80 mM KCl or higher. Next, we performed disassembly assays in which bundles were disrupted by changes in the solution conditions (Fig. 4, *B* and *C*). The scattering intensity at both pH 5 and 7 decreased as the KCl concentration increased (Fig. 4*B*), indicating that the bundles were disrupted by increasing ionic strength. We also performed disassembly assays by changing the pH. The scattering intensities remained unchanged after the pH was shifted to 6 or 7. However, when the pH was shifted to 8 or 9, the scattering intensities decreased to the background level within 2 min (Fig. 4*C*). These results indicate that SpeMreB5 bundle formation requires electrostatic interactions. To elucidate the structural basis of the electrostatic interaction dependence of bundles, we measured the aspect ratio of bundles (width/length) over a range of the KCl concentration (Fig. 4*D*). For both pH 5 and 7, the aspect ratio decreased as the KCl concentration increased, suggesting that the lateral interactions are more dominated by electrostatic interactions than the longitudinal interactions. For all tested KCl concentrations, the aspect ratio at pH 7 was higher than that at pH 5, suggesting that the lateral interactions at pH 7 are stronger than those at pH 5.

### The positively charged C-terminal region of SpeMreB5 is involved in promoting lateral interactions for bundle formation and aggregation at neutral pH

Each *Spiroplasma* MreB5 possesses a C-terminal unstructured region with a positive net charge (3, 4, 16). To investigate the effects of this region on bundle formation, we prepared two SpeMreB5 variants with truncations of 9 and 26 residues at the C terminus (ΔC9 and ΔC26, respectively) (Fig. 5*A*). SpeMreB5 ΔC9 is comparable to the previously used ΔC10 variant of SciMreB5, which has an excess Arg residue at the C terminus compared with SpeMreB5 (Fig. S2*A*) (2, 3), and SpeMreB5 ΔC26 is a variant in which all C-terminal residues outside the MreB folding domain are removed. ΔC9 was successfully purified. In contrast, ΔC26 did not bind to the Ni^2+^-NTA affinity column, suggesting that the 6 × His-tag was occluded in the ΔC26 variant. Moreover, the solubility of ΔC9 at pH 5 was not high enough for polymerization experiments, although the solubility at pH 7 was sufficient. Therefore, we analyzed the bundle formation of the C-terminal-truncated variant of SpeMerB5 using ΔC9 at pH 7. The initial intensity of ΔC9 measured by light scattering was the background level, unlike the wildtype (WT) (Figs. 3*A* and 5*B*), indicating that the initial aggregation of SpeMreB5 (Fig. 3*C*), which caused the high initial intensity, was formed *via* the C-terminal region. ΔC9 in the presence of Mg-ATP assembled slower than that of WT and did not reach a plateau within 15 min (Fig. 5*B*). However, the steady-state intensities of ΔC9 were only slightly lower than those of WT over the KCl concentrations (Fig. 5*C*). The aspect ratio of ΔC9 bundles was two times lower than those of WT (Figs. 5*D* and S1*T*), indicating that the ΔC9 bundles were thinner than the WT ones. To evaluate whether these features were caused before the bundle formation phase, we performed sedimentation assays under the conditions used in our previous study in which SpeMreB5 formed sheets but did not form bundles (2). Using these assays, we estimated the critical concentrations that reflect the minimum concentration required for polymerization and the ratio of the dissociation and association rates (Fig. S4, *A*, *B* and *E*). Those of WT and ΔC9 were not significantly different (Table 1), suggesting that truncation of the C-terminal nine residues did not affect the dynamics of polymerization and sheet formation. These results indicated that the C-terminal region of SpeMreB5 is involved in promoting lateral interactions for bundle formation.

**Table 1:** Bulk critical concentrations of SpeMreB5 WT with varying divalent cation conditions and ΔC9 variant measured using a sedimentation assay^a^

Condition	Cc of SpeMreB5 (μM)	*p*-value v.s. 2 mM Mg-ATP WT
2 mM Mg-ATP WT	0.09 ± 0.01	-
2 mM Mg-ATP ΔC9	0.11 ± 0.04	0.45
2 mM Ca-ATP ΔC9	0.09 ± 0.08	0.95
2 mM ATP (X2-free) WT	0.05 ± 0.03	0.07

The row below the critical concentrations indicates the *p*-value against the critical concentration in the presence of 2 mM Mg-ATP supported by Student’s *t-*test, showing that the significance of critical concentration differences is not supported for all tested pairs.

a The values are indicated as mean ± SD from three repeated measurements.

The scattering intensity of ΔC9 in the absence of Mg-ATP increased immediately after starting the measurement, and its assembly rate was much faster than that in the presence of Mg-ATP (Fig. 5*B*). This result suggests that SpeMreB5 undergoes different reaction paths with and without nucleotides. Moreover, the scattering intensity of ΔC9 in the absence of Mg-ATP plateaued at the same timescale as the WT in the absence of Mg-ATP (Fig. S3*J*), suggesting that the reaction path of WT and ΔC9 in the absence of Mg-ATP is identical. However, the plateau intensity of ΔC9 in the absence of Mg-ATP was half that of WT in the absence of Mg-ATP (Fig. 5*B*). ΔC9 at this condition formed bundles with lengths shorter than 1 μm (Fig. S1*T*). These results suggest that the aggregation formation as observed in WT is involved in forming large bundles in the absence of Mg-ATP.

### Bundle formation of SpeMreB5 requires Mg^2+^ at neutral pH but is inhibited by divalent cations at an acidic pH

Previous studies have revealed that bundle formation of actin superfamily proteins requires divalent cations (27, 28, 31, 32). We then examined the requirement of divalent cations for SpeMreB5 polymerization and the formation of higher-order structures. First, we performed sedimentation assays on SpeMreB5 under various divalent cation conditions (Fig. S4). The pellet amounts of SpeMreB5 were mostly constant over Mg^2+^ and Ca^2+^ concentrations (Fig. S4, *F*–*H*). The critical concentrations of SpeMreB5 were not significantly different among conditions in the presence of 2 mM ATP (divalent cation-free), Mg-ATP, and Ca-ATP (Fig. S4, *A* and *C*–*E*; Table 1). These results indicate that SpeMreB5 does not require divalent cations for polymerization or sheet formation.

We also examined the effects of divalent cations on SpeMreB5 bundle formation. At pH 7, in the presence of 2 mM Ca-ATP, SpeMreB5 formed sheets, while the corresponding condition in the presence of Mg-ATP formed bundles (Figs. 6*A* and S1*C*). In contrast, bundles were formed at pH 5 in the presence of 2 mM Ca-ATP (Fig. S5*A*) as well as in the presence of Mg-ATP (Fig. 1*A*). In the divalent cation-free condition at pH 7, most of SpeMreB5 formed sheets (Fig. 6*B*) and a few fractions formed bundles (Fig. S5*B*). Surprisingly, under divalent cation-free conditions at pH 5, amorphous aggregates were observed instead of filamentous structures (Fig. 6*C*).

To clarify the effects of divalent cations on the assembly dynamics of bundles, we performed time-course measurements of SpeMreB5 bundle assembly (Figs. 6, *D* and *E* and S5, *C* and *D*). In the divalent cation-free condition at pH 5, the scattering profile was single phase and plateaued at the same timescale as the first phase in the presence of Mg^2+^, indicating that the first phase of assembly dynamics at pH 5 reflects the aggregation of SpeMreB5 (Fig. 6, *C* and *D*). The plateau intensities of both the first and second phases in the presence of Mg^2+^ at pH 5 decreased as Mg^2+^ concentration increased. In particular, the first phase was indistinguishable in the presence of 3 mM and 5 mM Mg^2+^. Moreover, the assembly rate of the second phase decreased in an Mg^2+^-dependent manner (Fig. 6*D*). Ca^2+^ showed similar effects to Mg^2+^ on SpeMreB5 bundle formation at pH 5, while its inhibition efficiencies were less than that of Mg^2+^ (Fig. S5*C*). These results suggest that SpeMreB5 bundle formation and the ATP-induced aggregation at pH 5 are inhibited by divalent cations.

In contrast, the assembly dynamics of bundles at pH 7 showed puzzling Mg^2+^ dependence. Under divalent cation-free conditions, the lag phase continued for approximately 3 min and the intensity reached a plateau in approximately 17 min. Notably, as we performed the time-course measurements under a KCl concentration of 10 mM, which is lower than the EM observations, the scattering intensity was high, even though there were few bundle structures under the EM observation (Figs. 6E and S5*B*). In the presence of 1 to 3 mM Mg^2+^, the times for the lag phase and reaching the plateau were five and two times shorter, respectively, and the plateau intensities were two times higher than those in the divalent cation-free condition. The plateau intensity in the presence of 5 mM Mg^2+^ was not different from those in the presence of 1 to 3 mM Mg^2+^, whereas the lag phase became slightly longer (Fig. 6*E*). We also examined the effects of Ca^2+^ on bundle assembly at pH 7. Bundle assembly was suppressed in the presence of Ca^2+^. In particular, the intensity did not substantially increase within 20 min in the presence of 5 mM Ca^2+^ (Fig. S5*D*). These results indicate that SpeMreB5 bundle formation at pH 7 requires Mg^2+^.

Next, we measured steady-state intensities over a range of Mg^2+^ concentrations (Fig. 6*F*). We set the KCl concentration to 50 mM for consistency with EM observations. At pH 5, the scattering intensities decreased in a Mg^2+^-dependent manner and reached the detection limit at Mg^2+^ concentrations of 5 mM or higher (Fig. 6*F*, ocher). Of note, this result may overrate the Mg^2+^ effects on bundles, as the scattering intensities are likely derived from both bundle and aggregated structures (Figs. 1*A* and 6*C*). However, it is plausible that both aggregation and bundle formation were inhibited by Mg^2+^ at pH 5. At pH 7, the scattering intensities peaked in the presence of 3 mM Mg^2+^ and were nearly the background level at 0 and 15 mM Mg^2+^ (Fig. 6*F*, green), suggesting that the bundle formation efficiency at pH 7 is determined by the balance of the Mg^2+^ requirement for bundle formation and ionic strength effects. We also evaluated the bundle disassembly by increasing the concentration of divalent cations (Figs. 6*G* and S5*E*). At pH 5, the decrease in the scattering intensity upon the addition of MgCl_2_ and CaCl_2_ was greater than that by KCl with the same ionic strength (when the degree of dissociation is assumed to be 1 for all salts) (ocher lines in Figs. 6*G* and S5*E*). This phenomenon is common for CaCl_2_ addition at a pH of 7. In contrast, the decrease in the scattering intensity by MgCl_2_ at pH 7 was less than that by KCl (green lines in Figs. 6*G* and S5*E*), consistent with the Mg^2+^ requirement for bundle formation at pH 7 (Fig. 6, *E* and *F*). Disassembly assays were also performed by adding EDTA (a chelating agent for multivalent cations) at pH 7. However, the decrease in the scattering intensity by EDTA was only slightly different from that of a buffer with a pH identical to that of the EDTA solution (Fig. S5*F*), suggesting that Mg^2+^ was occluded in the bundles and was not accessed by the EDTA. Altogether, our results demonstrate that SpeMreB5 bundle formation at pH 5 is inhibited by divalent cations but that at pH 7 requires Mg^2+^.

## Discussion

**Figure 7:**
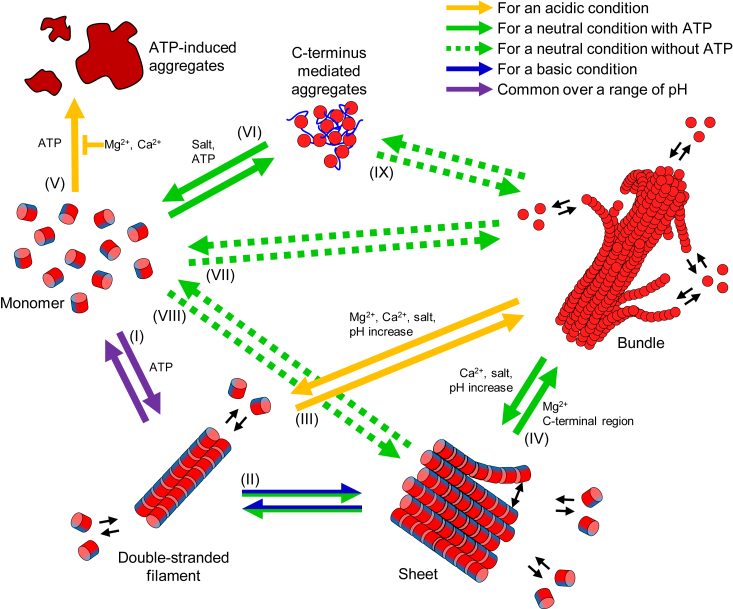
**Summary for SpeMreB5 polymerization.** The relationship among the six states found in this study (monomer, double-stranded filament, sheet, bundle, C-terminus-mediated aggregate, and aggregate induced by ATP) is suggested. An SpeMreB5 subunit is indicated by a *red circle* or a cylinder colored with *red* and *blue*. The positively charged unstructured C-terminus is shown as a *blue line* for the state of C-terminus-mediated aggregate. Reactions specific for acidic, neutral, and basic pH conditions in the presence of nucleotides are shown in solid arrows colored with *orange*, *green*, and *blue*, respectively. Reactions common over a range of pH are indicated using purple solid arrows. Reactions specific for conditions in the absence of nucleotides are shown in *green dotted lined arrows*. Factors that promote a reaction step are shown alongside each arrow. An inhibition factor against a reaction step is indicated by an arrow with a blunted end.

In this study, we investigated sheet and bundle formation of SpeMreB5 using EM and bulk biochemical assays. The transitions of the six states were observed in this study (Fig. 7). We discuss the properties of each state and their possible roles in *Spiroplasma* swimming in some states.

### Polymerization and sheet formation

ATP was required for polymerization at a low SpeMreB5 concentration and at acidic and basic pH, although SpeMreB5 at pH 7 formed filamentous structures under nucleotide-free conditions (Fig. 1). These results indicate that ATP promotes SpeMreB5 polymerization (Fig. 7-(I)), confirming our previous finding that nucleotide binding induces polymerization of SpeMreB3 and SpeMreB5 (2). SpeMreB5 formed double-stranded filaments at acidic pH and sheets at neutral and basic pH (Fig. 1*C*). Our previous study revealed that SpeMreB5 sheets are composed of an antiparallel double-stranded filament at one edge and a parallel alignment of protofilaments (2). The minimum unit of SpeMreB5 filamentous structures that we found was the double strand as well as the findings in a previous study (Figs. 1*C* and S1, *A* and *B*) (3), suggesting that sheet formation likely initiates from the double-stranded filaments (Fig. 7-(II)). The SciMreB5 protofilament is mostly surrounded by positively charged regions on both the front and back sides at pH 5, while the back side is negatively charged at pH 7 and 9 (Fig. 2, *A*–*C*), suggesting that sheet formation at an acidic pH is inhibited by electrostatic repulsion between the front and back sides. This is consistent with the results, in which SpeMreB3 at pH 7 did not form sheets (Fig. S1, *H* and *I*) and is surrounded by positively charged regions on both the front and back sides (Fig. S2*E*). Considering the protofilament orientations within the sheets, the same protofilament side (Fig. 2, *D*–*F*) faces the cytosolic region (2). This alignment indicates that SpeMreB5 sheets possess a wide negatively charged region at one surface.

### Bundle formation

SpeMreB5 formed bundles under low-ionic-strength conditions at acidic and neutral pH (diamonds in Fig. 1*C*). While this structure has not been observed in *Spiroplasma* cells (20, 21), it possibly reflects the properties of SpeMreB5 because there are clear differences in the tendency of bundle formation for SpeMreB3 and SpeMreB5 (Figs. 1*C* and S1, *H*–*J*). The bundle formation mechanisms at acidic and neutral pH are likely different, as the bundles showed distinct divalent cation dependences at these two pH conditions (Fig. 6, *D*–*G*). Under acidic conditions, in which sheet formation was inhibited (Fig. 1*C*), bundles likely grew from double-stranded filaments as nuclei (Fig. 7-(III)). The surface potential map of the SciMreB5 protofilament at pH 5 showed that the cytosolic side was negatively charged and the front side, which was exposed to the solvent when the double-stranded filaments were formed, was positively charged (Fig. 2, *A* and *D*), suggesting that these sides interact with each other to form bundles. The lengths of the bundles reached several micrometer orders, while those of the double-stranded filaments were submicrometer orders (Figs. 1*A* and S1, *A* and *B*), indicating that lateral interactions within bundles stabilize each protofilament.

In contrast, SpeMreB5 formed sheets at neutral pH prior to bundle formation in the presence of Mg-ATP (Figs. 3, *D* and *E* and S3*H*), suggesting that the sheets work as nuclei of bundles (Fig. 7-(IV)). As the steady-state intensities of ΔC9 were only slightly different from those of the WT (Fig. 5*C*), the C-terminal region was probably not involved and the negatively charged cytosolic region (Fig. 2*E*) was likely involved in the interactions for bundle formation. The positively charged front side was the sole candidate interaction partner for the negatively charged cytosolic region (Fig. 2*B*). The dependence of the front side on bundle formation is also supported by the inability of bundle formation at pH 9 (Fig. 1*C*). The negatively charged region of the subdomain IIA moiety at pH 9 became wider than that at pH 7 (Fig. 2, *B* and *C*), possibly leading to electrostatic repulsion for the inhibition of bundle formation. Although the front side is exposed to the solvent when SpeMreB5 forms sheets, it is unreasonable that the bundles are formed by sheet stacking, considering the tight packing of protofilaments within the bundles (Fig. 1*A*). Instead, it is most plausible that single protofilaments elongate on negatively charged surfaces, such as the cytosolic side of the sheets, facing the front side to grow into bundles. This model is consistent with previous findings in which SpeMreB5 and SciMreB5 do not only form interprotofilament interactions for antiparallel double-stranded filaments (2, 3, 4). Although the C-terminal unstructured region is expected not to be involved in interactions for bundle formation (Fig. 5*C*), this region was involved in promoting lateral interactions for bundle formation (Fig. 5, *B* and *D*), suggesting that nonspecific interactions *via* the C-terminal region increase the local concentration of SpeMreB5 around sheets and bundles to promote their assemblies. This idea is consistent with a previous study in which engineered proteins with flexible tubulin-binding regions on the outside of microtubules induced suprastructural formation such as microtubule doublets and branched microtubules (38). Bundle formation at neutral pH required Mg^2+^ and was inhibited by Ca^2+^ (Figs. 6, *E* and *F* and S5*D*), suggesting that SpeMreB5 has binding sites specific for Mg^2+^. These regions probably localize on the surface of the SpeMreB5 protofilaments as those for the bundle formation of actin filaments are on its surface (32). Inhibition of bundle formation by Ca^2+^ has also been reported for EcMreB (29), suggesting that Mg^2+^-binding sites for bundle formation are negatively charged regions common between SpeMreB5 and EcMreB, such as the moieties of subdomains IIA and IIB on the back side of the protofilament. Bundle formation was inhibited by divalent cations at acidic pH (Figs. 6*D* and S5*C*), suggesting that the putative Mg^2+^-binding regions at neutral pH turn their charges by pH shifts between 5 and 7.

Bundle formation dependent on divalent cations has also been reported for actin filaments. However, the optimal concentration of divalent cations for actin bundle formation is 10–50 mM, which is approximately 10 times higher than the optimal concentration for SpeMreB5 bundle formation (1–5 mM) (Fig. 6*F*) (32). Actin bundles are formed by bridging divalent cations with nine acidic residues (32), suggesting that electrostatic interactions are less involved in actin bundle formation than in SpeMreB5 bundle formation. This likely explains why actin bundles are resistant to the presence of high concentrations of divalent cations, which are high enough to disrupt SpeMreB5 bundles (Fig. 6*F*). Moreover, actin filaments adopt a right-handed helix, which can restrict interfilament interactions to form bundles. In contrast, the SpeMreB5 sheets are not helical (Figs. 1, *B* and *E* and S1, *D*–*G*), suggesting that small amounts of Mg^2+^ will affect bundle formation, as interprotofilament interactions for bundle formation are unlikely to be restricted.

### Aggregations induced and disassembled by ATP

We also found two SpeMreB5 aggregates that showed distinct responses to ATP (Figs. 3*C* and 5*C*). One was formed in the presence of ATP at acidic pH (Figs. 6, *C* and *D*, 7-(V), S5*C*). This aggregation is surprising because, to the best of our knowledge, aggregations dependent on ATP have not been reported for the actin superfamily proteins. The other was formed at a neutral pH *via* the C-terminal region (Figs. 3*C* and 5*B*). Considering the disaggregation by ionic strength increase (Figs. 3*B* and S3, *A* and *B*), this aggregation is likely formed by electrostatic interactions *via* the positively charged C-terminal region (Fig. 5*A*). The disassembly efficiency of Mg-ATP was higher than that of other salts with comparable ionic strengths (Figs. 3*B*, 7-(VI), S3, *A* and *B*). We cannot rule out the possibility that this aggregation was affected by the initial conditions of our experiments (5 × concentration prior to the assays, also described in Fig. 3). However, disassembly of aggregate by ATP is intriguing because, to the best of our knowledge, a comparable phenomenon has not been reported for actin. Although only the nucleotide-binding pocket has been reported for the ATP-binding site of MreBs (2, 3, 4, 5, 6), this site is unlikely to dominate the disaggregation phenomenon. ATP is not only known as a molecular unit of currency in life but also reported as a hydrotrope of proteins (39). ATP as the hydrotrope binds nonspecifically to the termini and loops of proteins (40). This is likely the case for SpeMreB5.

### SpeMreB5 polymerization in the absence of nucleotides

We found that SpeMreB5 polymerized in the absence of nucleotides at a neutral pH when the protein concentration was sufficiently high (Figs. 1, *D*–*F* and S1*S*). This finding is intriguing because the polymerization of SpeMreB5 and SciMreB5 has been thought to require ATP (2, 3, 4). The lag phase of ΔC9 in the nucleotide-free condition was indistinguishable, unlike that in the presence of Mg-ATP (Fig. 5*B*), suggesting that SpeMreB5 under nucleotide-free conditions undergoes reaction paths to directly shift the states from monomer to a structural state in the steady state (Fig. 7-(VII) and (VIII)). SpeMreB5 WT, which forms aggregate mediated by the C-terminus (Fig. 3*C*), in the absence of nucleotides reached a plateau intensity higher than that at ΔC9 without any intensity drops (Fig. 5*B*), suggesting the following two possibilities: (1) there is a reaction path from the aggregate to bundles (Fig. 7-(IX)) and (2) the number of bundle nuclei is limited by the C-terminal-mediated aggregation in WT, leading to growing each bundle larger than that of ΔC9 (Figs. 1*D* and S1*U*) despite their identical assembly rates (Fig. S3*J*).

### Biological significances of these structures

In this study, we have analyzed biochemical properties of SpeMreB5 *in vitro*. The properties observed in this study may contribute to the roles of SpeMreB5 in *Spiroplasma* swimming, although *in vivo* analyses will be required to confirm them. We revealed that the cytosolic region of SpeMreB5 is mostly charged negative (Fig. 2, *D*–*F*). Considering the subunit alignments within the sheet (2), SpeMreB5 sheets probably face the negatively charged surface to the cytosolic region when they bind to the inner side of the membrane through its C-terminal region (3). Assuming that the MreB sheet between a fibril bundle and the cell membrane is composed of MreB5 as observed by EM (20), it can be hypothesized that the MreB5 sheet interacts with fibril filaments *via* the wide negatively charged regions on the cytosolic side of the sheets (Fig. 2, *D*–*F*). Consistent with this idea, a previous study reported that SciMreB5 interacts with fibril (4). We also found that the positively charged unstructured C-terminal region was involved in promoting lateral interactions of bundle formation (Fig. 5). As the C-terminal region of SciMreB5 is involved in binding to the negatively charged *Spiroplasma* membrane (3), the property for promoting the lateral interaction may induce MreB5 filament stability on the *Spiroplasma* membrane.

## Conclusions

In this study, we clarified the interactions in SpeMreB5 sheets and the formation mechanisms of bundles followed by sheet formation (Fig. 7) by focusing on ionic strength and pH dependence (Fig. 4), lateral interactions promoted by the C-terminal region (Fig. 5), and distinct divalent cation dependences (Fig. 6). These findings will aid in the understanding of the molecular properties of SpeMreB5. In this study, we found two aggregation modes of SpeMreB5, with distinct responses to ATP (Figs. 3*C* and 6*C*). This finding also sheds light on the protein aggregation phenomena.

## Experimental procedures

### SpeMreBs expression and purification

SpeMreB3 and SpeMreB5 and its ΔC variants were expressed and purified as described (2). In brief, SpeMreB3 or SpeMreB5 was expressed in *E. coli* BL21 (DE3) or C43 (DE3) carrying *spemreB3* or *spemreB5* fused pCold-15b for 24 h at 15 °C with induction conditions of OD_600_ = 0.4 and IPTG = 1 mM. Cell pellets from 1 L of culture were resuspended in 20–40 ml of buffer A (50 mM Tris-HCl pH 8.0, 300 mM NaCl, 50 mM imidazole-HCl pH 8.0), sonicated, centrifuged, and purified using a HisTrap HP column (Cytiva) and a HiLoad 26/600 Superdex 200 pg. column (Cytiva) equilibrated with buffer B (20 mM Tris-HCl pH 8.0, 300 mM NaCl) at 4 °C.

### SpeMreB polymerization

For the sedimentation assays, SpeMreBs were polymerized with buffer S (20 mM Tris-HCl pH 8.0, 1 M NaCl, 200 mM L-Arginine-HCl pH 8.0, 5 mM DTT, 2 mM MgCl_2_, and 2 mM ATP) (2), which inhibited polymerization and amorphous aggregation under nucleotide-free conditions, unless otherwise stated. The solution conditions for other experiments are shown in the figure legend. The pH 5, 7, and 9 conditions were mediated by 50 mM CH_3_COOH-KOH pH 4.9, HEPES-KOH pH 7.0, and CHES-KOH pH 9.4, respectively, unless otherwise stated. All samples tested in this study contained 5 mM DTT to prevent unfavorable oligomerization of SpeMreBs *via* S-S bonds. Prior to polymerization, the SpeMreB buffer was exchanged from buffer B to the desired buffer in the absence of DTT, divalent cation salts, and ATP by an overnight dialysis at 4 °C. SpeMreBs with a concentration lower than the desired concentration were concentrated using an Amicon Ultra 10 K (Merck). Samples with the desired SpeMreB concentration were centrifuged (20,000*g* at 4 °C for 10 min) to remove aggregate, and DTT, MgCl_2_, and ATP were added to initiate polymerization. For nucleotide-free conditions, divalent cation salts and ATP were excluded from the buffer.

### Electron microscopy

As described (2), a sample (4 μl) was placed onto a 400-mesh copper grid coated with carbon for 1 min at room temperature (24–27 °C), washed with 10 μl water, stained for 45 s with 2% (w/v) uranyl acetate, air dried, and observed under a JEOL JEM-1010 transmission electron microscope at 80 kV, equipped with a FastScan-F214T CCD camera (TVIPS). The aspect ratio of the SpeMreB5 bundle was estimated from its length and width measured by the distance measurement tool of ImageJ (National Institutes of Health; http://rsb.info.nih.gov/ij/).

### Estimation of surface potential map

The surface potential maps were calculated with the PDB2PQR (41, 42) server using the PARSE forcefield (43, 44) in conjunction with PROPKA (45, 46, 47) to assign the protonation state at the provided pH conditions (48). Calculations were performed for each subunit, excluding the bound ligands. For the calculation of SpeMreB3 (PDB:7E1G), in which lysine residues were dimethylated prior to crystallization (2), the dimethylated lysine residues were replaced with the most probable rotamers of lysine in Dunbrank’s rotamer library (49) because the positive charge of a dimethylated lysine residue is weaker than that of unmethylated lysine. The surface potentials were visualized using Chimera ver 1.13.1 (50) with a color gradient from −10 kcal/mol/e (red) to +10 kcal/mol/e (blue).

### Sedimentation assay

SpeMreBs in buffer S polymerized at room temperature with a volume of 200 μl were polymerized for 1 to 6 h and centrifuged (100,000 rpm at 23 °C for 120 min) in a TLA-100 rotor (Beckman Coulter) (2). The pellet was resuspended in 200 μl water. The supernatant and pellet fractions were subjected to electrophoresis on a 12.5% Laemmli gel and stained with Coomassie Brilliant Blue R-250 to analyze the concentration of each fraction. The band intensities of SpeMreBs were quantified using ImageJ. The concentrations of the supernatant and pellet fractions were estimated as the products of the total SpeMreB concentration and the ratio of each fraction to the sum of the supernatant and pellet fractions.

### Static light scattering

Ninety-degree perpendicular light scattering experiments were carried out using FP-6200 (JASCO) in a single cuvette containing 60 μl sample solution at 25 °C under the control of a temperature stabilizer. Both the excitation and emission wavelengths were set to 650 nm. For the time-course measurements, a sample with protein and buffer concentrations five times higher than the desired was kept on ice prior to the assay, diluted in a solution at room temperature to mediate the composition, and immediately applied to the measurements. We defined t = 0 as the time point when the solution was set to the desired condition with a lag time of approximately 5 s due to manual mixing. For steady-state light scattering measurements, SpeMreB5 was polymerized in a microtube at room temperature for more than 2 h, which was long enough to reach a steady state, and mixed immediately before transferring to the cuvette. The baseline was set as the scattering intensity of water. Ionic strength (*IS*) was estimated using the following equation:

(1) $$IS=\frac{1}{2}\sum_{i}c_{i}z_{i}^{2}$$

where *c*_*i*_ and *z*_*i*_ are the concentration of the ion species and the charge of the ion, respectively.

## Data availability

Raw data are available from the corresponding author on reasonable request.

## Supporting information

This article contains supporting information (2, 36, 49).

## Conflict of interest

The authors declare that they have no conflicts of interest with the contents of this article.
