# Supplementary material for: Assembly properties of bacterial actin MreB involved in Spiroplasma swimming motility
Source: J Biol Chem. 2023 May 5;299(6):104793. doi: 10.1016/j.jbc.2023.104793 (PMC10279915; doi:10.1016/j.jbc.2023.104793)
Supplement: Supporting Figures S1–S5 [file mmc1.pdf]

Assembly properties of bacterial actin MreB involved in *Spiroplasma* swimming motility

Daichi Takahashi[1], Makoto Miyata[1,2], Ikuko Fujiwara[1,2,3,*]

[1]Graduate School of Science, Osaka Metropolitan University, Osaka, Japan.

[2]The OMU Advanced Research Center for Natural Science and Technology, Osaka Metropolitan University, Osaka, Japan

[3]Department of Materials Science and Bioengineering, Nagaoka University of Technology, Nagaoka, Niigata, Japan

* To whom correspondence should be addressed:

Ikuko Fujiwara: ikukofujiwara@vos.nagaokaut.ac.jp

Figure S1. Negative-staining EM observation of SpeMreB3 and SpeMreB5 over various pH and ionic strength. (**A-G**) Negative-staining EM images of 10 μM SpeMreB5 polymerized with 2 mM Mg-ATP at pH (**A-B**) 5, (**C-E**) 7, and (**F-G**) 9 with KCl concentrations of (**C**) 50, (**A, D,** and **F**) 200, and (**B, E,** and **G**) 400 mM. (**H-L**) Negative-staining EM images of 10 μM SpeMreB3 polymerized with 2 mM Mg-ATP at pH (**K**) 5, (**H-J**) 7, and (**L**) 9 with KCl concentrations of (**H** and **K-L**) 50, (**I**) 200, and (**J**) 400 mM. (**M-R**) Negative-staining EM images of (**M-N**) 10 and (**R**) 5 μM SpeMreB5 and (**O-Q**) 10 μM SpeMreB3 incubated in the absence of Mg-ATP at pH (**M** and **O**) 5, (**P** and **R**) 7, and (**N** and **Q**) 9 with (**M-Q**) 50 and (**R**) 200 mM KCl. (**S**) Negative-staining EM image of 10 μM SpeMreB5 polymerized in the absence of Mg-ATP in previously defined standard buffer (composed of 20 mM Tris-HCl pH 7.5 and 100 mM KCl) (1). (**T-U**) Negative-staining EM image of 10 μM SpeMreB5 ΔC9 polymerized (**T**) in the presence and (**U**) in the absence of 2 mM Mg-ATP at pH 7 with (**T**) 50 and (**U**) 10 mM KCl. Scale bars are indicated in each panel.

Figure S2. Electrostatic natures of MreB family proteins. The inter-protofilament interaction surface for the antiparallel filament formation is defined as “back”, and the opposite side is defined as “front.” (**A**) Space-filling model of SciMreB5 (PDB: 7BVY) highlighting the residues different from those of SpeMreB5. The residues that are negatively charged, positively charged, and uncharged in SpeMreB5 but have a different charged state in SciMreB5 are colored red, blue, and orange, respectively, and highlighted as “the residue in SpeMreB5 / that in SciMreB5.” The residues that

differ each other between SpeMreB5 and SciMreB5 in the same charged state are colored yellow. The other residues, which are the same in SpeMreB5 and SciMreB5 are colored gray. Owing to the sequence difference between SpeMreB5 and SciMreB5, the net charge of SpeMreB5 is 1 lower than that of SciMreB5. (**B-G**) Surface potential maps of MreB family proteins. The coulombic electrostatic potential is indicated by a color gradient from blue (10 kcal/mol/e) to red (−10 kcal/mol/e); namely blue, white, and red regions indicate positively charged, uncharged, and negatively charged regions, respectively. The structural models of double-stranded filament were created by fitting four MreB molecules to each subunit of a double-stranded filament structure of CcMreB (PDB: 4CZJ). (**B-D**) Surface potential maps on the membrane side of double-stranded filament model of SciMreB5 AMPPNP (PDB: 7BVY) at pH (**B**) 5, (**C**) 7, and (**D**) 9. The positions of facing subdomains (IA and IB) are labeled for the left side subunits. A circle with dashed line indicates the region where is possibly occupied by the positively charged C-terminus, which is not visualized in the reference structure. (**E-G**) Surface potential maps of protofilaments and double-stranded filament models of (**E**) SpeMreB3 (PDB: 7E1G), (**F**) TmMreB (PDB: 1JCG), and (**G**) EcMreB (the monomer was modeled by AlphaFold2 (2)) at pH 7. The protofilament model of EcMreB (front and back side views) were created by fitting two EcMreB molecules to each subunit of a protofilament of a CcMreB double-stranded filament (PDB: 4CZJ). A circle with dashed line on the membrane side of SpeMreB3 double-stranded filament model indicates the region that is possibly occupied by the amphipathic N-terminal helix, which is not visualized in the

reference structure. Lysine residues of SpeMreB3 are di-methylated (1), weakening the positive charge of lysine residues. Therefore, the di-methylated lysine residues were replaced with the most probable rotamers of lysine in Dunbrank's rotamer library (3).

Figure S3. Bundle formation and disaggregation dynamics of SpeMreB5. For time-course light scattering, data are shown as mean ± S.D. from three independent measurements. (**A**) Intensity decreases of SpeMreB5 by additives with corresponding ionic strengths with 2 mM Mg-ATP (assuming the degree of dissociation as 1, see Experimental procedures). Minimum intensities of light scattering traces for 75 s with the indicated additives added to 10 μM SpeMreB5 at pH 7 with 10 mM KCl were subtracted from those without additives. Bars indicate S.D. from three repeated measurements. Symbols indicate *p*-value supported by Student's *t*-test (* $p < 0.05$, ** $p < 0.01$). (**B**) Initial dynamics of 10 μM SpeMreB5 at pH 7 with 10 mM KCl by adding 2 mM Mg-ATP (green), 30 mM KCl (gray), 30 mM NaCl (blue), 7.5 mM $MgCl_2$ (brown), 7.5 mM $CaCl_2$ (orange), and 7.5 mM Na_2SO_4 (light green) with the measurement time for 20 s which was long enough to reach the minimum intensity for the condition adding 2 mM Mg-ATP. Of note, the ionic strengths of these additives are approximately the same assuming the degree of dissociation as 1 for all salts (see Experimental procedures). (**C-D**) Negative-staining EM images of 10 μM SpeMreB5 polymerized with 2 mM (**C**) Mg-AMPPNP and (**D**) Mg-ADP at pH 7 with 50 mM KCl. Scale bars are indicated in each panel. (**E**)

Assembly dynamics of 10 μM SpeMreB5 in the presence of 2 mM Mg-ATP (green), Mg-AMPPNP (light green), and Mg-ADP (orange) at pH 7 with 10 mM KCl. The spectra of the first 30 s are highlighted in the inset. (**F**) Dynamics of SpeMreB5 bundle formed in the absence of nucleotides. SpeMreB5 with 10 μM concentration was assembled in the absence of nucleotides at pH 7 with 10 mM KCl (dotted line on the left graph). After several min, 2 mM Mg-ATP was added, and time-course light scattering was measured (solid line in the right graph). (**G**) Assembly dynamics of 10 μM SpeMreB5 polymerized with 2 mM Mg-ATP at pH 7 with 10 (green) and 20 (light green) mM KCl measured by light scattering. (**H**) Negative-staining EM of 10 μM SpeMreB5 polymerized for 2 min at pH 7 with 20 mM KCl in the presence of 2 mM Mg-ATP. The image was taken in another field of the same EM grid as in Fig. 3D. Scale bar is indicated in the panel. (**I**) Unnormalized plots of Fig. 4A which shows steady-state light scattering of 10 μM SpeMreB5 polymerized with 2 mM Mg-ATP over a range of KCl concentrations at pH 5 (ocher) and 7 (green). Bars indicate S.D. from three independent measurements. (**J**) Normalized traces of SpeMreB5 WT (green dotted line) and ΔC9 (purple dotted line) in the absence of Mg-ATP shown in Fig. 5B.

Figure S4. Sedimentation assays of SpeMreB5. Each sample was incubated in buffer S, centrifuged, and analyzed using SDS-PAGE, as previously described (1). All reactions were conducted in the presence of 2 mM ATP. For divalent cation-free conditions, 1 mM EDTA-NaOH pH 8.0 was added to avoid effects from

contaminating the amounts of multivalent cations. (**A-D**) Sedimentation assays of SpeMreB5 (**A** and **C-D**) WT and (**B**) ΔC9 polymerized with 2 mM (**A-B**) Mg-ATP, (**C**) Ca-ATP, and (**D**) ATP (divalent cations free) over the range of SpeMreB5 concentration. (**E**) Quantified precipitation amounts of sedimented SpeMreB5. Precipitated SpeMreB5 was resuspended in water equivalent amount to the sample, and the resulting concentrations were plotted over the total SpeMreB5 concentrations with linear fitting. Error bars indicate S.D. from three repeated measurements. Critical concentrations were estimated as the x-intercept of each linear fit and summarized in Table 1. (**F-G**) Sedimentation assay of 3 μM SpeMreB5 over the range of (**F**) $MgCl_2$ and (**G**) $CaCl_2$ concentrations. (**H**) Quantified precipitation amounts of sedimented SpeMreB5. Precipitated SpeMreB5 was resuspended in water equivalent amount to the sample, and the resulting concentrations were plotted over the divalent cation concentrations with linear fitting. Error bars indicate S.D. from three repeated measurements.

Figure S5. Divalent cation dependence of SpeMreB5 bundle. For time-course light scattering, data are shown as mean ± S.D. from three independent measurements. For divalent cation-free conditions, 1 mM EDTA-NaOH pH 8.0 was added to avoid effects from contaminating amounts of multivalent cations. (**A-B**) Negative-staining EM images of 10 μM SpeMreB5 polymerized in the presence of (**A**) 2 mM Ca-ATP at pH 5 and (**B**) 2 mM ATP at pH 7. KCl concentration was constant for 50 mM KCl. The image of the panel B was taken at another field of the

same EM grid as that for Fig. 6B. Scale bars are indicated in each panel. (**C-D**) Ca^{2+}-dependent assembly dynamics of 10 μM SpeMreB5 at pH (**C**) 5 and (**D**) 7 with 10 mM KCl measured using light scattering. Polymerization was initiated by adding 2 mM ATP with varying $CaCl_2$ concentrations as indicated in the color scales in the panels. (**E**) Unnormalized light scattering traces of Fig. 6G that shows disassembly dynamics of SpeMreB5 bundles induced with divalent cations. (**F**) Time-course light scattering measurements of SpeMreB5 bundles disassembly induced by 10 mM HEPES-KOH pH 8.1 (gray) and EDTA-NaOH pH 8.0 (green). The initial bundle solutions were prepared by polymerizing 10 μM SpeMreB5 in the presence of 2 mM Mg-ATP in 10 mM HEPES-KOH pH 7.0 with 40 mM KCl. The measurement in which the buffer composition was unchanged are indicated with a dark green line.

References

1. Takahashi, D., Fujiwara, I., Sasajima, Y., Narita, A., Imada, K., and Miyata, M. (2022) ATP-dependent polymerization dynamics of bacterial actin proteins involved in *Spiroplasma* swimming. *Open Biol* **12**, 220083

2. Mirdita, M., Schütze, K., Moriwaki, Y., Heo, L., Ovchinnikov, S., and Steinegger, M. (2022) ColabFold: making protein folding accessible to all. *Nature methods* **19**, 679-682

3. Shapovalov, M. V., and Dunbrack, R. L. (2011) A smoothed backbone-dependent rotamer library for proteins derived from adaptive kernel density estimates and regressions. *Structure (London, England : 1993)* **19**, 844-858

Fig S1

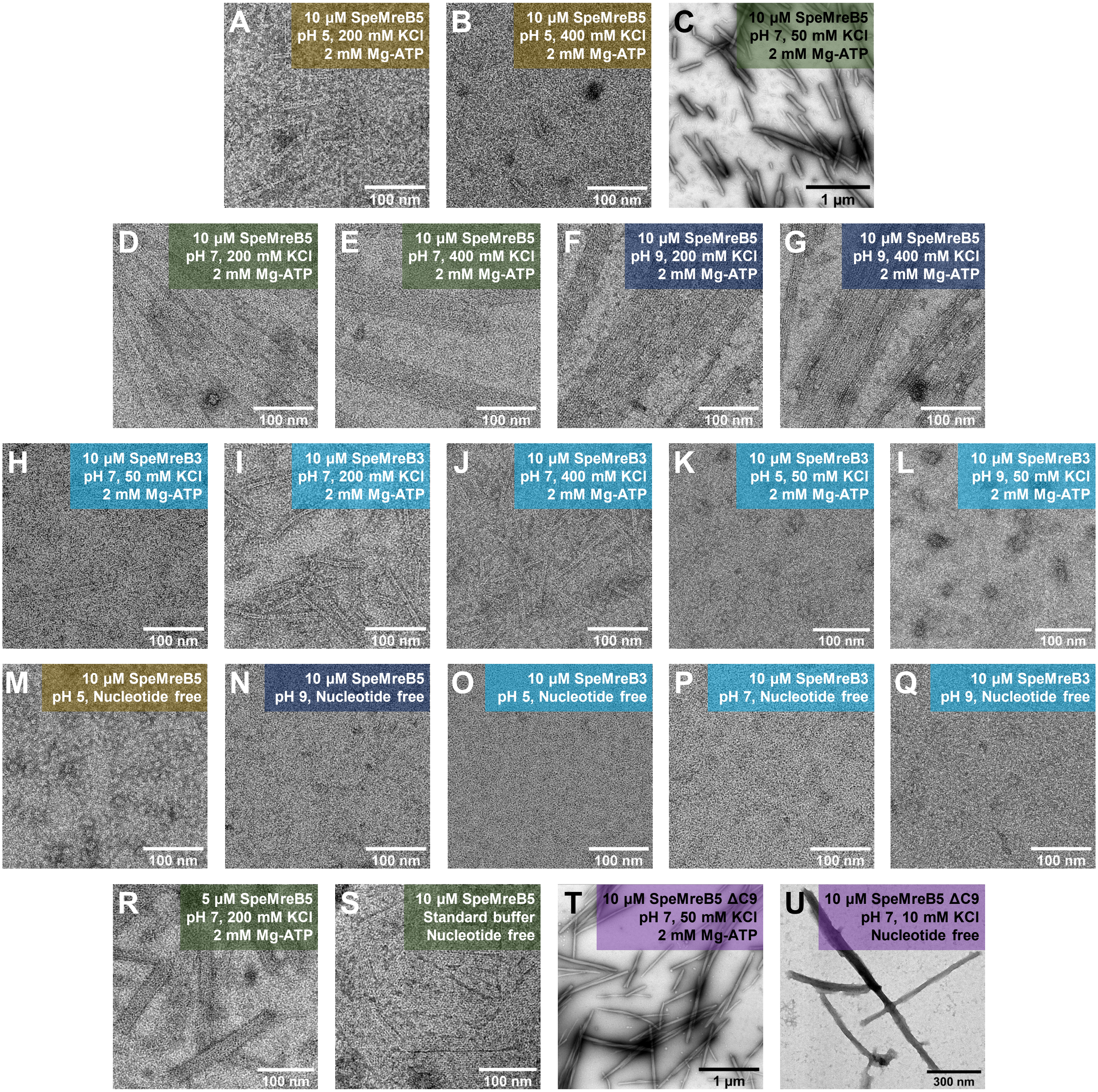
A
10 µM SpeMreB5 pH 5, 200 mM KCl 2 mM Mg-ATP
100 nm
B
10 µM SpeMreB5 pH 5, 400 mM KCl 2 mM Mg-ATP
100 nm
C
10 µM SpeMreB5 pH 7, 50 mM KCl 2 mM Mg-ATP
1 µm
D
10 µM SpeMreB5 pH 7, 200 mM KCl 2 mM Mg-ATP
100 nm
E
10 µM SpeMreB5 pH 7, 400 mM KCl 2 mM Mg-ATP
100 nm
F
10 µM SpeMreB5 pH 9, 200 mM KCl 2 mM Mg-ATP
100 nm
G
10 µM SpeMreB5 pH 9, 400 mM KCl 2 mM Mg-ATP
100 nm
H
10 µM SpeMreB3 pH 7, 50 mM KCl 2 mM Mg-ATP
100 nm
I
10 µM SpeMreB3 pH 7, 200 mM KCl 2 mM Mg-ATP
100 nm
J
10 µM SpeMreB3 pH 7, 400 mM KCl 2 mM Mg-ATP
100 nm
K
10 µM SpeMreB3 pH 5, 50 mM KCl 2 mM Mg-ATP
100 nm
L
10 µM SpeMreB3 pH 9, 50 mM KCl 2 mM Mg-ATP
100 nm
M
10 µM SpeMreB5 pH 5, Nucleotide free
100 nm
N
10 µM SpeMreB5 pH 9, Nucleotide free
100 nm
O
10 µM SpeMreB3 pH 5, Nucleotide free
100 nm
P
10 µM SpeMreB3 pH 7, Nucleotide free
100 nm
Q
10 µM SpeMreB3 pH 9, Nucleotide free
100 nm
R
5 µM SpeMreB5 pH 7, 200 mM KCl 2 mM Mg-ATP
100 nm
S
10 µM SpeMreB5 Standard buffer Nucleotide free
100 nm
T
10 µM SpeMreB5 ΔC9 pH 7, 50 mM KCl 2 mM Mg-ATP
1 µm
U
10 µM SpeMreB5 ΔC9 pH 7, 10 mM KCl Nucleotide free
300 nm

Fig S2

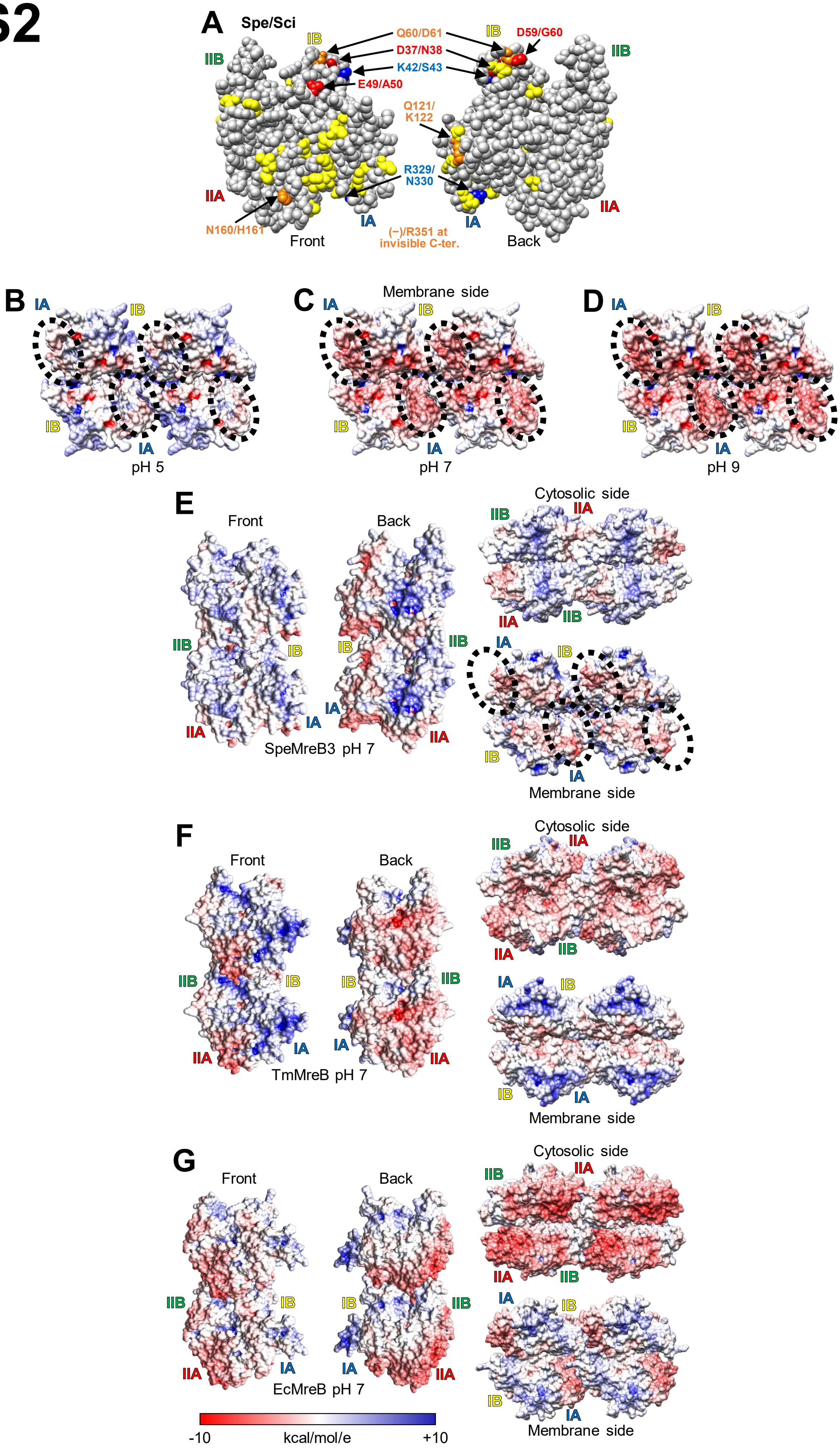
A
Spe/Sci
Q60/D61
D59/G60
D37/N38
K42/S43
E49/A50
Q121/
K122
R329/
N330
N160/H161
(−)/R351 at
invisible C-ter.
Front
Back
IA
IB
IIA
IIB
B
C
D
Membrane side
pH 5
pH 7
pH 9
E
Front
Back
Cytosolic side
SpeMreB3 pH 7
Membrane side
F
Front
Back
Cytosolic side
TmMreB pH 7
Membrane side
G
Front
Back
Cytosolic side
EcMreB pH 7
Membrane side
-10
kcal/mol/e
+10

Fig S3

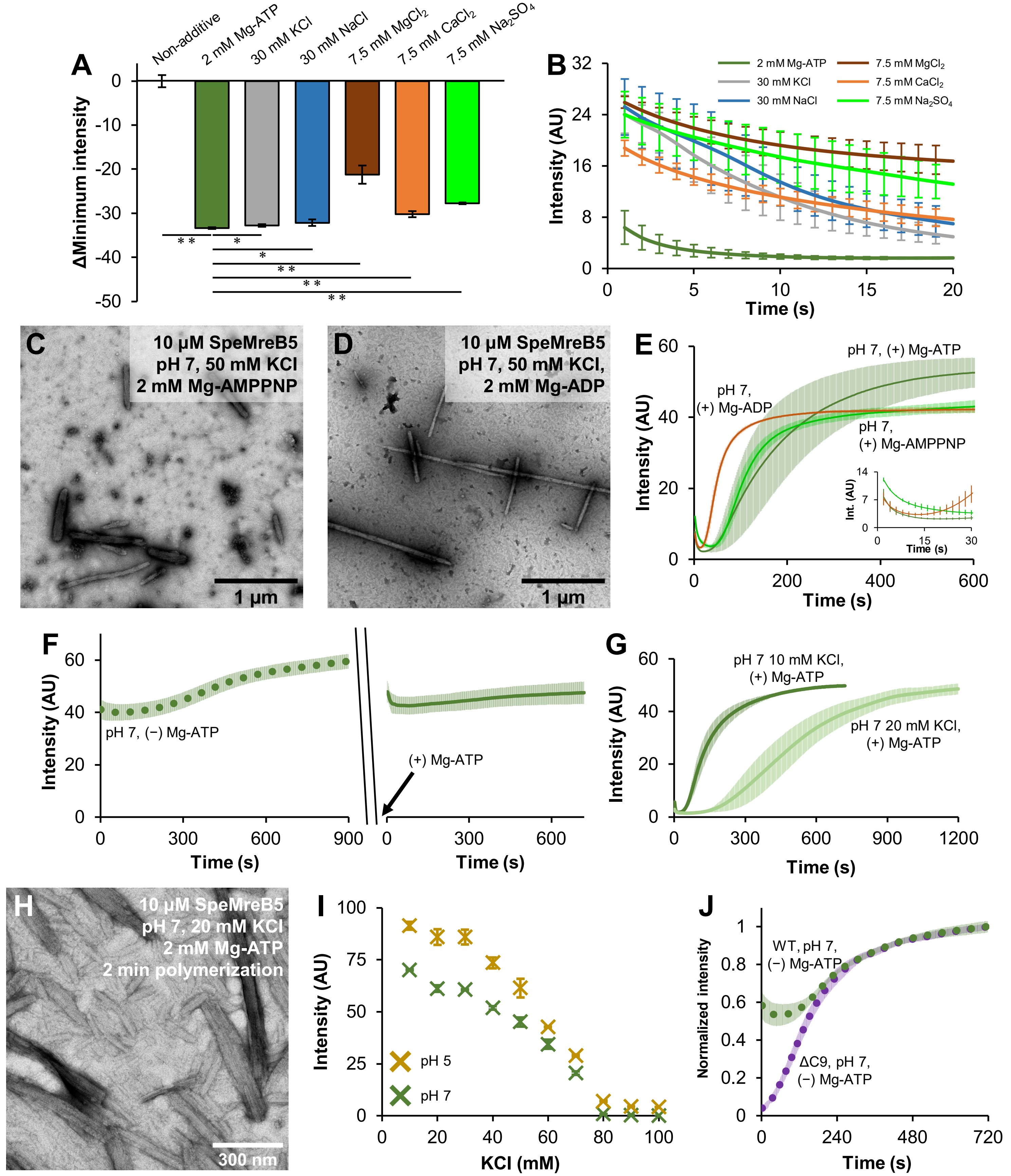

A
Non-additive
2 mM Mg-ATP
30 mM KCl
30 mM NaCl
7.5 mM MgCl2
7.5 mM CaCl2
7.5 mM Na2SO4
ΔMinimum intensity
B
2 mM Mg-ATP
30 mM KCl
30 mM NaCl
7.5 mM MgCl2
7.5 mM CaCl2
7.5 mM Na2SO4
Intensity (AU)
Time (s)
C
10 µM SpeMreB5
pH 7, 50 mM KCl
2 mM Mg-AMPPNP
1 µm
D
10 µM SpeMreB5
pH 7, 50 mM KCl,
2 mM Mg-ADP
1 µm
E
pH 7, (+) Mg-ATP
pH 7,
(+) Mg-ADP
pH 7,
(+) Mg-AMPPNP
Intensity (AU)
Int. (AU)
Time (s)
F
pH 7, (−) Mg-ATP
(+) Mg-ATP
Intensity (AU)
Time (s)
G
pH 7 10 mM KCl,
(+) Mg-ATP
pH 7 20 mM KCl,
(+) Mg-ATP
Intensity (AU)
Time (s)
H
10 µM SpeMreB5
pH 7, 20 mM KCl
2 mM Mg-ATP
2 min polymerization
300 nm
I
pH 5
pH 7
Intensity (AU)
KCl (mM)
J
WT, pH 7,
(−) Mg-ATP
ΔC9, pH 7,
(−) Mg-ATP
Normalized intensity
Time (s)

Fig S4

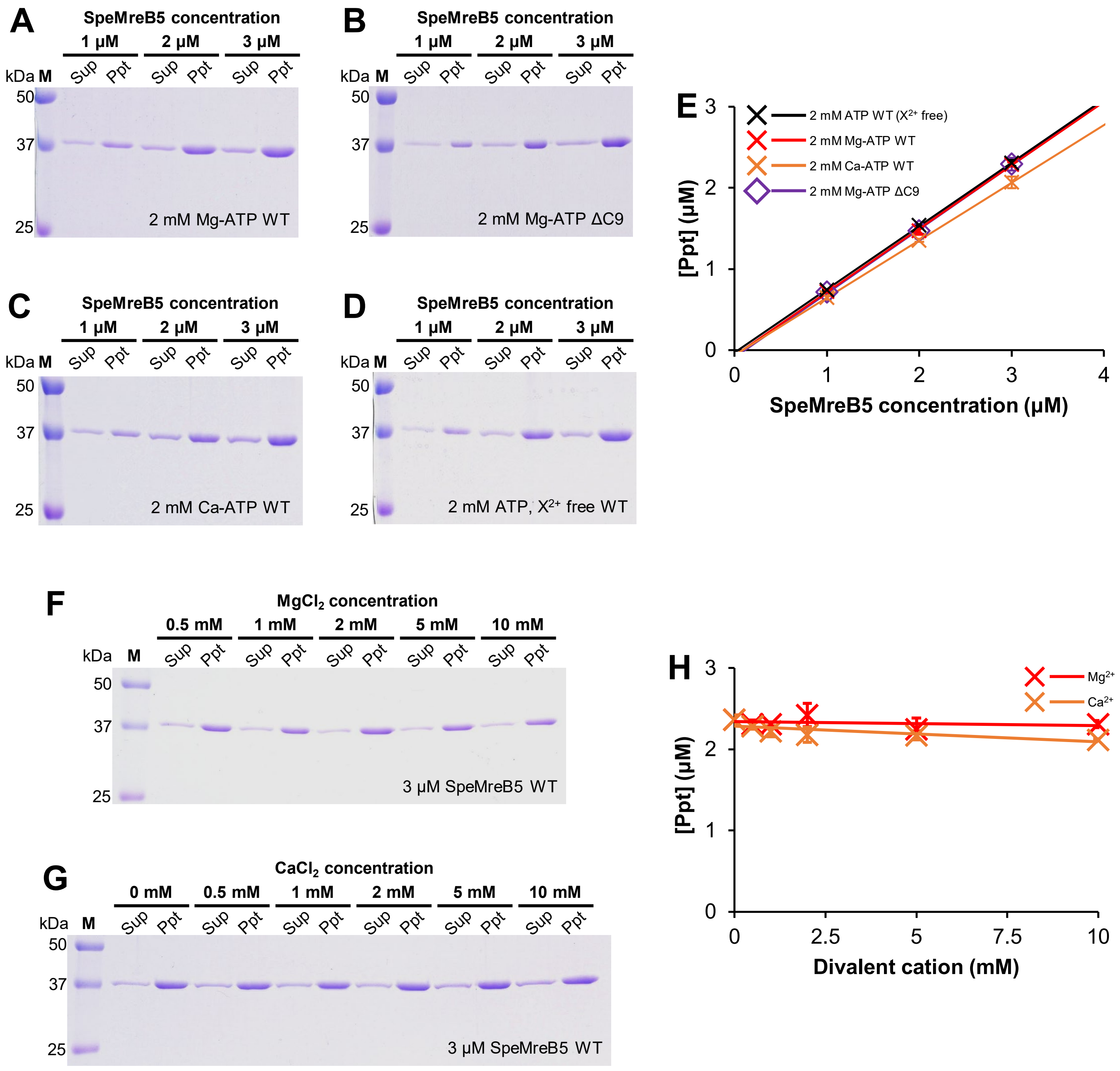
A
SpeMreB5 concentration
1 µM
2 µM
3 µM
kDa M
Sup
Ppt
50
37
25
2 mM Mg-ATP WT
B
2 mM Mg-ATP ΔC9
C
2 mM Ca-ATP WT
D
2 mM ATP, X2+ free WT
E
2 mM ATP WT (X2+ free)
2 mM Mg-ATP WT
2 mM Ca-ATP WT
2 mM Mg-ATP ΔC9
[Ppt] (µM)
SpeMreB5 concentration (µM)
F
MgCl2 concentration
0.5 mM
1 mM
2 mM
5 mM
10 mM
3 µM SpeMreB5 WT
G
CaCl2 concentration
0 mM
H
Mg2+
Ca2+
Divalent cation (mM)

Fig S5

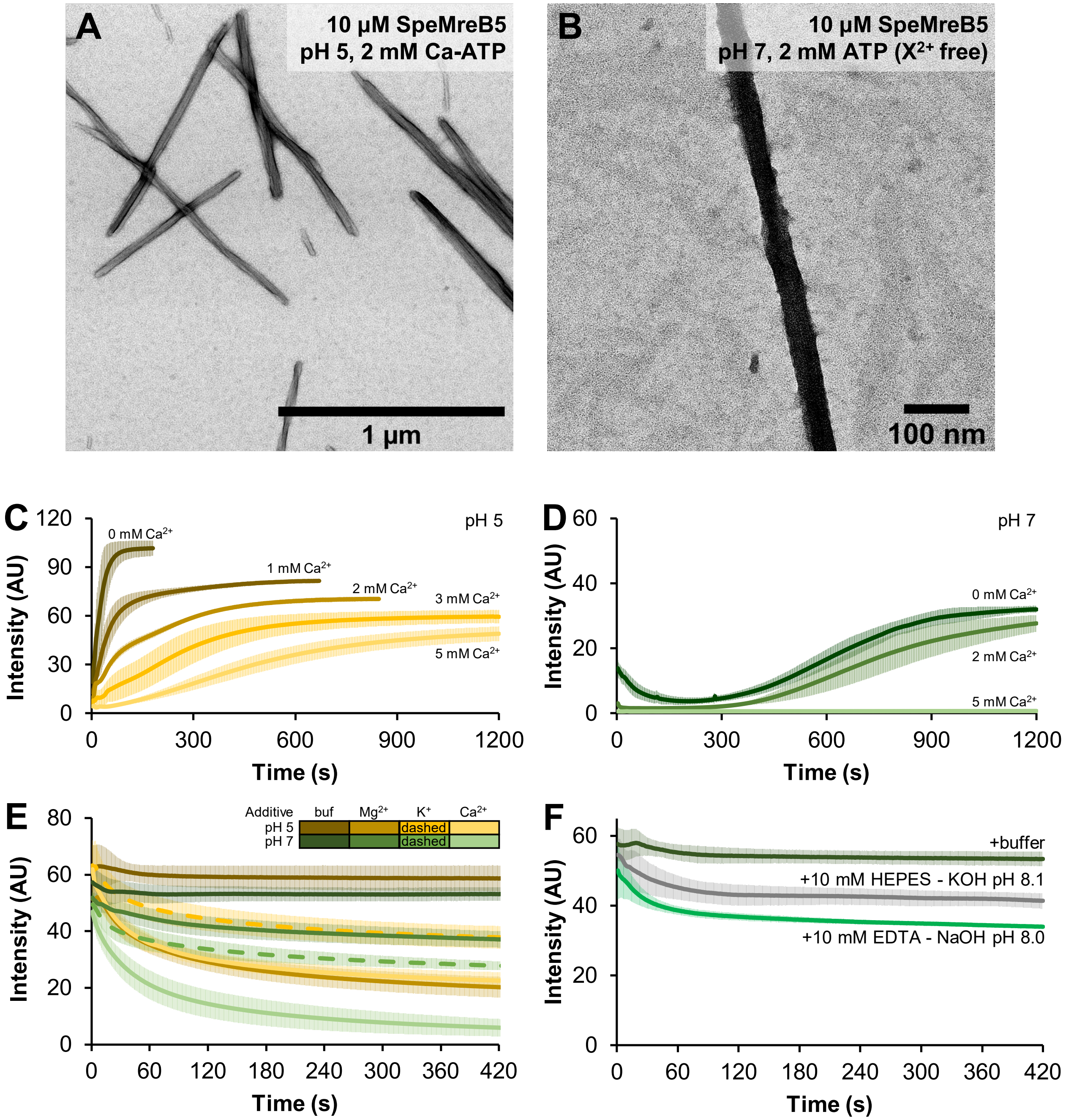
A
10 µM SpeMreB5
pH 5, 2 mM Ca-ATP
1 µm
B
10 µM SpeMreB5
pH 7, 2 mM ATP (X2+ free)
100 nm
C
pH 5
0 mM Ca2+
1 mM Ca2+
2 mM Ca2+
3 mM Ca2+
5 mM Ca2+
Intensity (AU)
Time (s)
D
pH 7
0 mM Ca2+
2 mM Ca2+
5 mM Ca2+
Intensity (AU)
Time (s)
E
Additive
buf
Mg2+
K+
Ca2+
pH 5
pH 7
dashed
dashed
Intensity (AU)
Time (s)
F
+buffer
+10 mM HEPES - KOH pH 8.1
+10 mM EDTA - NaOH pH 8.0
Intensity (AU)
Time (s)
